# Supplementary material for: Determinants of late detection and advanced-stage diagnosis of breast cancer in Nigeria
Source: PLoS One. 2021 Nov 3;16(11):e0256847. doi: 10.1371/journal.pone.0256847 (PMC8565753; doi:10.1371/journal.pone.0256847)
Supplement: Attachment — Submitted filename: Response to Review comment August 7 2021.docx [file pone.0256847.s002.docx]

We thank the reviews and editor for taking the time and effort to review and suggest corrections in our manuscript. The suggestions have greatly improved the manuscript.

Kind regards

| **Reviewer 1** | comment | Review made in Manuscript | Location in manuscript |
| --- | --- | --- | --- |
| 1 | Spelling and grammar corrections | Spelling, grammar, and punctuations reviewed throughout the article | Throughout the article |
|  | Use of “And” to begin sentence not appropriate | Sentence reviewed | manuscript line 64) |
| 2 | Results need re-working to make tables more understandable | Result section and all tables reviewed to simplify | Results section |
|  | Why are odds ratio presented for only a few variables in table 2 | Odds ratio presented only for variables with statistically significant analysis. However, the odds ratio has been moved to prose section of the result to maintain uniformity | Manuscript line 156-169 |
|  | Use of letters better replaced with actual headings in tables | Letters replaced with actual headings describing each subsection in tables | In tables |
|  | Presenting of the p-values not uniform | p-values presented uniformly through out the article | Results section |
|  | What is the risk of progression, how did you arrive at that percentage | Progression is the number progressing from early to an advanced stage before diagnosis  Risk of progression is number progressing divided by the total number (N) | Description inserted in footnote of table 4 |
|  | What the legend in page 22 mean | They are just legends of the tables, they have been removed |  |
| **Reviewer 2** |  |  |  |
| 1 | Percentages missing in tables | Percentages included where appropriate in all tables |  |
| 2 | Fischer’s exact should be corrected as Fisher’s exact | Corrected | Manuscript line 129 |
| 3 | Present forest plots for the OR and RR values in tables 3 and 4 | Forest plots presented as figures 1 and 2 |  |
| **Journal requirements** |  |  |  |
|  | Review reference list | Reference list reviewed and corrected; retracted manuscript not used |  |
|  | Ensure manuscript conforms with journal style | Headings reviewed to conform to journal style |  |
|  | Make data available | Minimum data available in excel spread sheet |  |
|  | Provide additional information about database used | The database was a local simple Microsoft database produced specially for the purpose of the original research | Manuscript line 94 |
|  | Give information on whether data was deidentified and if IRB gave waiver | De identified data was used and IRB approval was obtained | Manuscript lines 87 and 92 |
